# Nurse lecturers’ experiences with online teaching during the pandemic at a public university in Gauteng, South Africa

**DOI:** 10.4102/curationis.v45i1.2371

**Published:** 2022-11-30

**Authors:** Andile G. Mokoena-de Beer, Sophy M. Moloko

**Affiliations:** 1 Nursing Science Department, School of Health Care Sciences, Sefako Makgatho Health Sciences University, Pretoria, South Africa

**Keywords:** experiences, lecturer, nursing, online teaching, public university

## Abstract

**Background:**

Nurses’ training has been mostly face-to-face in the South African context. This mode of delivery was linked to producing nurses who are critical thinkers, problem solvers and competent in practical skills. However, the emergence of coronavirus disease 2019 (COVID-19) accelerated the need for online teaching in nursing. Nurse lecturers were forced to teach online in order to save the academic project, despite concerns about the competencies and calibre of nurses produced through online teaching.

**Objectives:**

This study aimed to explore and describe nurse lecturers’ experiences with online teaching during the COVID-19 pandemic at a public university in Gauteng, South Africa.

**Method:**

A qualitative, exploratory design was utilised. Six nurse lecturers – two males and four females – were purposefully selected to participate in this study. Data were collected through in-depth interviews to obtain rich, thick descriptions from the nurse lecturers who experienced online teaching. Content analysis was used to analyse the data.

**Results:**

Five themes emerged as, (1) challenges related to the learner management system; (2) challenges related to competency; (3) factors out of the span of control of the lecturer; (4) indirect benefits of online teaching; and (5) recommendations to facilitate the smooth delivery of online teaching.

**Conclusion:**

The findings established that nurse lecturers experienced challenges when teaching online, which resulted in frustrations and discomfort for lecturers.

**Contribution:**

The study revealed the challenges nurse lecturers faced while teaching online. It highlights the need for nurse lecturers to be trained and supported to enhance online teaching and learning.

## Introduction

Nurses are trained to provide healthcare to a group of patients with complex health problems through a holistic approach to nourish, cherish and provide curative care and treatment to the sick (Potter et al. 2023:89). Nurses are further trained to demonstrate compassionate care, described as understanding another’s pain with a commitment to doing something to relieve that pain (Hofmeyer et al. 2018). According to Potter et al. (2023:94), a nurse learns to deliver care artfully with compassion, care and respect for each patient’s dignity and personhood, and nurses’ training thus reflects these values.

This article reports on the contemporary issues in nursing education during the coronavirus disease 2019 (COVID-19), focusing on nurse lecturers’ experiences with online teaching. As a clinical course, nurses’ teaching is typically face-to-face to ensure learners understand the concept of compassionate care through the demonstration of practical skills. Contact teaching in nursing ensures that nurses’ training equips them with critical thinking skills, problem-solving skills and practical skills at a deeper level. However, there seems to be a change in nursing programmes as the need for online teaching increased rapidly during the COVID-19 pandemic (Broussard & Wilson 2018:40). Online teaching is a digital teaching method that is offered through a specified learner management system (LMS) and requires internet connectivity (Sadiku, Adebo & Musa 2018:74).

The selected university had introduced online teaching environments – even for nursing programmes – prior to the pandemic, demanding the use of both traditional face-to-face and online teaching approaches. This required nurse lecturers’ flexibility. However, the online teaching environment was typically only utilised for loading teaching and learning material (Teaching and Learning 2020:1).

Amid the COVID-19 social-distancing lockdown restrictions, the university in Gauteng was forced to use online teaching and learning for all courses (Nabolsi et al. 2021:828; Teaching and learning 2020:1). Online teaching required nurse lecturers to demonstrate the effectiveness of teaching online in order to produce nurses who will deliver nursing care in a compassionate manner (Richter & Schuessler 2019:28). However, as a clinical course, online teaching in nursing has increased concerns, especially in terms of the quality and calibre of nurses produced. The use of videos, graphics and other online cognitive aids as teaching strategies to prepare nurses for practice has been found to be central to online teaching (Markaki et al. 2020:39). However, these strategies appeared to have reduced the level of learner-to-teacher interaction. A study by Broussard and Wilson (2018:41) also highlighted a decline in the use of simulation; instead, nurse lecturers resorted to using videos as teaching tools as these were readily available and accessible. The use of videos as one of the teaching methods instead of face-to-face simulation impacts the quality and outcomes of nursing training because of minimal physical contact between lecturers–students and students–patients (Aziz et al. 2020:183; Broussard & Wilson 2018:41).

The move to online teaching also resulted in expert nursing educators grounded in traditional content delivery methods suddenly becoming novice online teachers in need of a new set of best practices (Authement & Dormire 2020:2). Regardless of the method of teaching, the social responsibility of professional nursing carries an imperative of excellence in nursing education to produce high-quality nurses (Authement & Dormire 2020:5). Therefore, nurse lecturers are expected to effectively utilise online teaching to deliver a nursing curriculum without compromising the quality of teaching and learning. It is crucial to understand how nurse lecturers experienced the transition from face-to-face to online teaching. Hence, the aim of the study was to explore and describe nurse lecturers’ experiences with online teaching during the COVID-19 pandemic at a public university in Gauteng, South Africa.

### Problem statement

The demand for online teaching in most universities is increasing and has been exacerbated by the emergence of the COVID-19 outbreak. The pandemic necessitated a paradigm shift from traditional face-to-face to online teaching. Nurse lecturers were left with no choice but to teach online because of the implemented lockdown restrictions during the outbreak. However, online teaching, especially in nursing, is being criticised for the lecturer’s lack of physical presence (Broussard & Wilson 2018:41).

The researchers work at a university and have observed with concern how online teaching in nursing is implemented. Ultimately, online teaching emerged as an alternative to ensure continuity in teaching and learning for nursing students. Studies across the world have explored how the move to online teaching has impacted nursing education as a clinical course and nursing students’ perspectives of online teaching (Broussard & Wilson 2018:41; Hofmeyer et al. 2018:311; Markaki et al. 2020:40). However, there is a paucity of research conducted in South African universities on how nurse lecturers experienced online teaching during the pandemic in a clinically oriented course. This motivated the researchers to conduct this study, and the following research question arose: What are nurse lecturers’ experiences with online teaching during COVID-19 at a public university in Gauteng, South Africa?

### Purpose of the study

The purpose of the study was to obtain an in-depth understanding of nurse lecturers’ experiences with online teaching during the COVID-19 pandemic at a public university in Gauteng, South Africa.

### Objective of the study

The study’s objective was to explore and describe nurse lecturers’ experiences with online teaching during COVID-19 at a public university in Gauteng, South Africa.

#### Definition of key concepts

*Experiences* are defined by the Oxford Popular School Dictionary (2008:141) as knowledge gained from doing or seeing things. In this study, experiences refer to the knowledge nurse lecturers gained from teaching online.

A *lecturer* is an educator involved in training people and enabling them to acquire knowledge and develop skills (Oxford Popular School Dictionary 2008:128). In this study, a nurse lecturer is an educator who conducts education and training for nurses enabling them to acquire knowledge and develop essential skills in the nursing field.

*Nursing* is a caring profession that involves interpersonal and interactional processes that assist individuals, families and communities to prevent or cope with the experience of illness or suffering (Kotzé 2017:7). In this study, nursing is a caring profession that includes educating and training individuals into the caring profession.

*On line teaching* – ‘online’ refers to being connected to or available through a system, while ‘teaching’ entails the guiding of studies and imparting of knowledge (Merriam-Webster Dictionary 2022:1). Therefore, online teaching in this study refers to guiding nursing studies and imparting knowledge through an electronic learner management system (LMS).

A *public university* is an institution that provides higher education on a full-time, part-time or distance basis and is established as a public higher education institution under the *Higher Education Act, 101 of 1997* (Department of Higher Education & Training 2021:114). In this study, a public university refers to a higher education institution that provides training for nurses.

## Research design and methods

A qualitative, exploratory design was utilised to explore and describe nurse lecturers’ experiences with online teaching during the COVID-19 pandemic at a public university in Gauteng, South Africa. The researchers chose a qualitative research design as it is a scholarly and rigorous approach used to describe life experiences from the perspective of the lecturers involved in online teaching (Babbie 2021:91; Gray & Grove 2021:75; Polit & Beck 2021:31).

### Study setting

The study was conducted at a university in Gauteng, situated in the northern part of Tshwane in Gauteng, South Africa. The university offers various healthcare programmes, including nursing. The nursing science department offers undergraduate nursing degrees for a minimum duration of four years and postgraduate courses ranging from 2 to 5 years.

### Population and sampling

The study population was nursing lecturers working at the nursing science department and engaged in facilitating both undergraduate and postgraduate programmes. Nineteen lecturers were employed in the Nursing Science Department at the time of the study. The lecturers were purposefully selected and deemed suitable to be included in the study (Bertram & Christiansen 2018:60). The selection criteria included nurse lecturers with a minimum teaching experience of three years and online teaching experiences during the COVID-19 pandemic. The sample comprised two male and four female nurse lecturers, aged 32–68 years, with an average of two years’ experience in online teaching. The sample size was determined by data saturation, where no new information emerged from the nurse lecturers (Brink, Van der Walt & Van Rensburg 2018:126).

### Recruitment process

The researchers gained access to the participants through the head of the nursing science department (HoD) after obtaining ethical clearance for the study. The participants were recruited following approval by the HoD. An email containing study information and a request for participation was sent to the nurse lecturers. Appointments to obtain informed consent and collect data were scheduled with the nurse lecturers who voluntarily agreed to participate in the study.

### Data collection

Data were collected through in-depth interviews, which took place at a convenient platform for the participants, including the online blackboard meeting space and face-to-face at the participants’ offices. These were carried out at a time convenient for the lecturers when they were not busy with teaching and learning activities. The interviews were conducted in English, and each interview lasted approximately 45–60 min. All interviews were audio-recorded with permission from the nurse lecturers. To ensure consistency, the researchers asked each nurse lecturer the same central question from the interview guide: ‘what is your experience of online teaching during the COVID-19 pandemic within a clinical course?’ Probing questions were used to obtain more information. These open-ended questions were formulated according to how the participants responded to the central question. Some of the probing questions were:

- How does this experience impact how you deliver your teaching?
- How does online teaching impact your role as a nurse lecturer?
- What challenges are you facing with online teaching as a nurse lecturer within a clinical course?
- How do you deal with the challenges you are facing with online teaching as a nurse lecturer?
- What positive impact has online teaching brought into nursing as a clinical course?
- In your view, what can be done to assist you, as a nurse lecturer, deal with the impact of moving to online teaching within a clinical course?

### Data analysis

A non-numeric examination and interpretation of the interviews were performed using content analysis to discover the underlying meanings and patterns of relationships from the data (Babbie 2021:385). Content analysis entails an analysis of data in the form of textual materials that involve coding data and classifying or categorising individual pieces of the data. In this study, content analysis followed five steps, as described by Babbie (2021:385): Step 1: select the content to be analysed. In this study, it was the transcribed interviews. Step 2: define the units and categories of analysis. The units of meaning were coded and categorised. Step 3: code the content. The rules for coding and organising the units into categories were set. Step 4: code according to the rules. Each text was examined, and all relevant data in the appropriate categories were recorded. Step 5: analyse the results and draw conclusions. Data were examined to find patterns, and conclusions were drawn in line with the research question.

### Measures to ensure trustworthiness

Trustworthiness refers to the degree of confidence researchers have in their data and analysis (Polit & Beck 2021:276). The following criteria were implemented to ensure the study’s trustworthiness. *Creditability* was ensured by prolonging engagement with participants and asking questions until data saturation was reached. This promoted trust and rapport that assisted the researchers to obtain accurate and rich information. The interviews were audio-recorded to allow accurate transcription of data. Member checking was applied by soliciting participants’ views on the researchers’ interpretation of the findings (Creswell & Poth 2018:255). To ensure *transferability*, the researchers generated rich and thick descriptions of the data by describing the methods of the study, including the participants and the setting in detail (Polit & Beck 2021:276). A log of all research processes followed during the study was kept, enhancing the study’s *dependability*. To ensure *confirmability*, the researchers reflected the participants’ voices, phrases, and the conditions of the inquiry, and not the researchers’ biases, motivations or perspectives (Polit & Beck 2021:276). All interviews were recorded to ensure an audit trail of raw data. *Authenticity* was ensured by recording field notes to convey the feeling and tone of nurse lecturers’ experiences (Polit & Beck 2021:277).

### Ethical considerations

The study was approved by Sefako Makgatho Health Sciences University Research Ethics Committee (reference number SMUREC/M/45/2021: IR). The principle of autonomy, as the right to self-determination, was adhered to. The nurse lecturers were informed about the purpose, benefits and risks associated with their participation in the study. Their participation was voluntary without any coercion. Written informed consent was obtained from the participants, and they were informed about the study and the implications of participating in the study. Anonymity was ensured by not using the nurse lecturers’ true identities when conducting the interviews, but assigning numerical codes to them. To promote confidentiality, none of the information given by the nurse lecturers was linked to them; thus, no private information was disclosed. Harm to participants was prevented by limiting any inconveniences during the study, especially humiliation during the interviews. All participants were treated fairly without any prejudice or discrimination.

## Results

Six nurse lecturers, ranging from 30 to 69 years, participated in the study. The participants consisted of two males (33.3%) and four females (66.7%), with at least one year’s experience in online teaching (see Table 1).

**TABLE 1:** Participants’ demographic profile (*N* = 6).

Category	Level	*N*
Gender	Male	2
	Female	4
Age in years	30–49	2
	50–69	4
Highest level of education	Master’s degree	3
	Doctoral degree	3
Years of experience as a nurse lecturer	0–9	2
	10–19	3
	20–29	1
Years of experience in online teaching	1–2	5
	3–4	1

Five themes emerged reflecting the nurse lecturers’ experiences with online teaching during the COVID-19 pandemic at a public university in Gauteng, South Africa. The themes were: (1) challenges related to the LMS; (2) challenges related to competency; (3) factors out of the span of control of the lecturer; (4) indirect benefits of online teaching and (5) recommendations to facilitate the smooth delivery of online teaching (see Table 2).

**TABLE 2:** Themes and sub-themes of lecturers’ experiences with online teaching.

Themes	Sub-theme
Theme 1: Challenges related to the LMS	Difficult to use
	Emergent transition to online teaching
	Time constraints
Theme 2: Challenges related to competency	Insufficient training
	Inability to transfer skills
Theme 3: Factors out of the span of control of the lecturer	Unstable internet connectivity
	Electrical power cut-offs
Theme 4: Indirect benefits of online teaching	Convenience and cost-effectiveness
	Improved teaching skills
Theme 5: Recommendations to facilitate the smooth delivery of online teaching	Training and support

### Theme 1: Challenges related to the learner management system

The data revealed that participants experienced teaching nursing online was difficult. Their challenges were linked to the use of the online system or platform. This meant that the nurse lecturers had to set aside time to acquaint themselves with the LMS and spend time preparing for teaching using the system. The nurse lecturers shared the following:

‘That was number one. I had no idea how the system works, so I needed to be taken through it. Uh, I’m going to say my experience at first was a very difficult experience. Because I have never had an opportunity to teach online until I came here.’ (Participant 2, 34-year-old male)

‘uuhm teaching a clinical course through online it is not simple … it was not easy but we are trying but uuhm we do not even think that the students are getting it the way they should get it.’ (Participant 1, 52-year-old female)

‘I would say it’s difficult it was very difficult to manage. Um, because you, you are an unable to simulate.’ (Participant 5, 32-year-old male)

The transition to online teaching was necessitated by the emergence of COVID-19, and was experienced by the nurse lecturers as abrupt as it had to occur concurrently with the teaching of students. The nurse lecturers expressed that they were learners and teachers at the same time. While they were learning to use the system, they had to teach using the system to save the academic project. They echoed:

‘It was a new phenomenon to start with, then to move from face to face to teaching online, Our training, even if we were orientated, it was not long enough. Because we were trained whilst working because we couldn’t stop the year to say now let’s First to go for training and then we’ll do online teaching. We would go for training now and then if we have a class next week you have to practice all those things.’ (Participant 1, 52-year-old female)

‘The system, the system itself, I had to sit down and actually be taught how to use the system for accessing all of these online classes. … And during this time, I also had to be in class. So, it was a, hit the ground running kind of moment for me because I had to learn and still do.’ (Participant 2, 34-year-old male)

‘From my side, I would think it started off a bit difficult because you had to learn how to utilize this new online system that you were, you were almost sort of forced to learn it right there and then, and know everything about it. Otherwise you, you, you get your, you’re not going to go into class. So that period of having to attend the course so that you can learn how the online learner management system works also uh … Um … became a bit of a factor because you had to be the teacher and be the student at the same time.’ (Participant 5, 32-year-old male)

The nurse lecturers’ time was mostly spent trying to design content suitable for the online teaching environment. Online teaching meant they spent more in a session than they would in a traditional face-to-face class; as a result, some of the classes went beyond the normal class period. This claim is supported by the following participant statements:

‘It was more time. It was challenging for me. It was a real challenge because I had to create some space in my busy schedule to get them to be here for face to face as well.’ (Participant 6, 54-year-old female)

‘I spent a lot of time doing, I’m just saying I spent a lot of time really not teaching, but planning for teaching planning, planning for assessments and correcting all of those challenges, or attending to all of the challenges related to the very teaching and the assessments. So, it was a drag. It wasn’t real drag.’ (Participant 2, 34-year-old male).

‘Took a lot of our time, because what I had to do is after the student presented what they uh … worked on as a group, I also had to come in and, um … close the gaps sort of like repeat what they were saying.’ (Participant 5, 32-year-old male)

### Theme 2: Challenges related to competency

The nurse lecturers echoed that they experienced various challenges, including insufficient training to teach online. This made them feel incompetent while delivering content, and they resorted to just teaching and not engaging with the students because of fear of using the system. The participants made the following comments:

‘So for the fact that I didn’t have the formal training and I had to depend on Google to take me through.’ (Participant 4, 57-year-old female)

‘Because there was no time for me to actually attend those formal lessons myself on how to use the system.’ (Participant 2, 34-year-old male)

The participants were also unable to transfer skills in the online platform (both soft skills and hard skills) because of limitations in interpersonal interaction. In instances where these skills were taught, it was not feasible to measure competency, be it in cognitive knowledge or clinical knowledge in an online environment. This was confirmed by statements such as:

‘So some of these skills that are interpersonal, like interpersonal relations may be missed in the process and I don’t think that skill I transferred it very well to say, did you check that you did 1, 2, 3? I think I concentrated mostly on the checklist.’ (Participant 1, 52-year-old female)

‘The application of knowledge. It is, it’s only very few of the students inform us that we’ll be able to.’ (Participant 3, 68-year-old female)

‘That transfer of knowledge, they are not going to absorb it directly from you because they have zone out … they struggled, to apply the knowledge to learned theory to the practical component of things.’ (Participant 2, 34-year-old male)

Nursing is a clinically oriented course where one needs to measure if learning has taken place. The nurse lecturers were unable to measure clinical skills competency as most of the teaching occurred online. They had to demonstrate skills online and students could not demonstrate back to the lecturers in an online platform:

‘And you want to demonstrate that to the students and also grant them an opportunity to ask questions and, um, practice at the same time after you’ve simulated. Now, when you are doing it online, it is not doable.’ (Participant 5, 32-year-old male)

Some of the nurse lecturers resorted to using other teaching methods such as pre-recorded videos, incorporating them into their online teaching. However, this was not easy for them as there were not enough relevant videos available to the nurse lecturers. If there were, most were not in the South African context:

‘You don’t have videos that are relevant that addressing the situation, the reality in South Africa, and remember also language is a barrier because some of them, it was in high English.’ (Participant 4, 57-year-old female)

‘We relied more on the, on the video. So basically, you would have to look for, for teaching aids in the form of videos, on either YouTube or any, um, because in the context of Africa, the difficulty is even if you rely on the videos, they are American standard most of them. We don’t have much of our own, and that will cover our dynamics as well. So, you would look at the video and then share the links with the student and say, please look at this video and note down questions if any, or anything that you might want me to clarify in class.’ (Participant 5, 32-year-old male)

‘Then I wouldn’t be doing the demonstrations practically. So now here, I had to rely on pictures and I had to use drawings of which I’m also not a very good drawer. I am not given artistically in that regard. So I had to also learn how to do that because some it’s very difficult for students to just follow the voices and I also had to look for a lot of videos.’ (Participant 2, 34-year-old male)

### Theme 3: Factors out of the span of control of the lecturer

The nurse lecturers experienced that some of the factors were beyond their control. They shared that these factors contributed to challenges in managing their online classes, thereby creating a disequilibrium in the online teaching environment. Unstable network coverage also seemed to be a general challenge and the nurse lecturers expressed the following:

‘The coverage, the internet coverage that could pose a problem. The technical in the sense that you would perhaps maybe struggle to do to upload something.’ (Participant 3, 68-year-old female)

‘They struggled with network because some are staying in villages whereby the connectivity becomes a problem for them.’ (Participant 6, 54-year-old female)

‘Other challenges because sometimes we found that the signals for the network are not good where you are. So it means that the class, they can be disturbed because you are unable to communicate well with the students.’ (Participant 1, 52-year-old female)

‘Yeah, there were always complaints about the network’s been down the Wi-Fi. The Wi-Fi not being there. And then that class really not being possible … There are network glitches that are going to be experienced, obviously by the students …’ (Participant 2, 34-year-old male)

Other challenges that were out of the nurse lecturers’ control included unplanned electrical power outages, which rendered their scheduled classes invalid. It affected their classes and, at a certain point, classes could not continue:

‘You find that even your learners also may say, ma’am, we cannot connect and sometimes there’s this load shedding of electricity. You find that this load shedding or the electricity just go off, or maybe there’s a cable theft in your area. It means your class is disturbed so online through all those challenges of just network also is still a challenge.’ (Participant 1, 52-year-old female)

‘We have been on a load shedding situation where it absolutely nothing to do now with the students themselves. They were trying to write the test and then suddenly there was load shedding.’ (Participant 2, 34-year-old male)

‘Sometimes you are teaching at home when there is load shedding connectivity – just cut off and the students are waiting for you to teach them.’ (Participant 6, 54-year-old female)

### Theme 4: Indirect benefits of online teaching

While teaching online was experienced as difficult, it came with indirect benefits for the nurse lecturers. This was indicated as a rather positive experience by the participants. Some of the benefits of online teaching were cited as convenience and cost-effectiveness, as supported by the following statements:

‘[*P*]ositive part is that you could reach them in large numbers, like the undergrad ones and is nice because sometimes you can stay at home, working from home without travelling at your own space without any interference … So at least that you would have save petrol for yourself and also you have saved much because don’t have to buy things outside.’ (Participant 6, 54-year-old female)

‘[*T*]he positive aspect of online was that if the network is good, you can still do it while at home or in Limpopo rather than coming to University were in you are teaching.’ (Participant 1, 52-year-old female)

‘And of course, the fact that I can teach the classes from home. I don’t have to go to the office if I’m having an online (Participant 5, 32-year-old male)

Good teaching skills are linked to effective class management. Therefore, during the online teaching process, the nurse lecturers learned new skills, which improved their teaching.

‘Yeah, you always have to be creative … it was a newly added skill on my part because I have always been very well comfortable with the traditional way of teaching. And now, this was a new skill that I had to adapt to. So online teaching also took me out of my comfort zone or recording itself. So, I had to also use really innovative teaching strategies. I had to be innovative.’ (Participant 2, 34-year-old male)

‘I think the positive in this is that, uh … we sort of had to learn, we are in 4IR any case. So we knew we had to learn how to be in path with the current trends of doing things.’ (Participant 5, 32-year-old male)

### Theme 5: Recommendations to facilitate smooth delivery of online teaching

The nurse lecturers expressed their views on how to better facilitate online teaching in clinically oriented courses. They echoed the need for sufficient training and support from the educational institution (university) in order to enhance their online teaching skills:

‘Yeah. I think the issue of training of lecturers online should be ongoing, because sometimes you find that there are new methods of doing things.’ (Participant 1, 52-year-old female)

‘We need to be assisted in order to realize that we can still reach these students, whether we are with them in the classroom or not. There are different ways in which we can do that.’ (Participant 2, 34-year-old male)

‘I think institutions of higher learning must give more support to the staff. This is what can be followed, and this is how you prove it. And also to give training like proper training … But if they, there is support in terms of knowledge translation, those who know the system. Sit us down and give us proper workshop.’ (Participant 5, 32-year-old male)

## Discussion

The aim of this study was to explore and describe nurse lecturers’ experiences with online teaching during the COVID-19 pandemic at a public university in Gauteng, South Africa. The discussion of the findings is based on five identified themes: challenges related to the LMS, challenges related to competency, factors out of the span of control of the lecturer, indirect benefits of online teaching and recommendations to facilitate the smooth delivery of online teaching.

It emerged from the study that nurse lecturers experienced challenges with the LMS, especially at the inception of online teaching. They found the system difficult to use because of their unfamiliarity with the system’s user interface. The majority of lecturers were not conversant with the technicality of the system because it was their first encounter with online teaching. Unfamiliarity with online educational applications and programmes could negatively influence the lecturers’ ability to effectively implement online teaching (Baroudi & Shaya 2022:12).

The sudden or unexpected transition from face-to-face to online teaching because of COVID-19 lockdown restrictions posed a challenge for the lecturers. Although the lecturers were orientated on the LMS, the transition process did not offer sufficient opportunity for preparation as it was abrupt and unplanned. The lecturers had to learn the system at the same time as teaching online because the university could not afford to lose educational time. The lack of preparedness on the LMS resulted in lecturers’ discomfort in using some of the tools that can enhance online teaching, because the nurse lecturers were not competent in using the system (Seetal, Gunness & Teeroovengadum 2021:209). To enhance the smooth transition to online teaching, Hayat et al. (2021:9) recommend that the LMS should be easy to use, lecturers should have the required abilities to use the system, and technical and troubleshooting support teams should be availed to them.

It was reported that more time is spent preparing and delivering the online teaching and learning activities compared with face-to-face teaching. Some of the time was spent fixing errors that occurred because of the unfamiliarity with the LMS. Hence, it is important for institutions to ensure that lecturers are conversant with the LMS prior to the implementation of online teaching.

The study revealed that the lecturers lacked competencies in online teaching strategies. However, the literature has documented appropriate instructional strategies for online teaching, which include the division of learning units into small chunks, increased students’ participation and interaction, voice and tone management, and blending online with offline self-learning (Bao 2020:115; Mahmood 2021:202). The lecturers in the study were not prepared on how to develop and deliver online lessons; it was a trial-and-error situation. They were uncertain about the effectiveness of the strategies they used in transferring knowledge and the application thereof. Their lack of competencies was attributed to a lack of formal training on the LMS and the online teaching strategies. Similarly, a study investigating teachers’ self-efficacy for online teaching found that teachers were not equipped to adapt the face-to-face curriculum to online platforms; they experienced challenges in choosing suitable online teaching strategies (Baroudi & Shaya 2022:12). The lack of training prior to engagement in the online context during the COVID-19 lockdown restrictions was thus not unique to this study. It was found to be a concern and influenced most teachers’ self-efficacy in implementing the online system (Baroudi & Shaya 2022:7).

Nursing is a clinical course that requires skill competencies. The learners need to be competent in soft skills, which include interpersonal skills, and hard skills, which can be physically observed. However, online teaching did not offer students the opportunity to develop interpersonal skills as part of their nursing skills. Nabolsi et al. (2021:834) affirm that online teaching and learning do not improve learners’ skills in teamwork, collaboration and positive attitudes because of a lack of lecturer–learner relationships and peer interaction. Although the lecturers devised online measures to demonstrate nursing skills to the students, they were unable to measure the students’ competency in the skills. Using videos to demonstrate some clinical skills was also a challenge because most of these were not relevant to the South African context. Hence, this study found online teaching to be less effective in transferring nursing skills to students. Saha et al. (2022:7) assert that online teaching is unsuitable for conducting and monitoring practical classes. Therefore, a blended approach is recommended (Zhu & Zhang 2022:2399).

Unstable internet connectivity is one of the infrastructural challenges that hinder online teaching and learning (Janse Van Rensburg 2018:78; Zhu & Zhang 2022:2398). Even though technical support was available from the university, it could not mitigate challenges related to unstable internet connectivity because it was out of their span of control. The challenge with internet connectivity and the bandwidth of the internet supply affected online teaching as some lecturers struggled to upload teaching material. This affected students’ access to learning, especially those who reside in areas with poor connectivity. The bandwidth challenge was similar to that mentioned in the study of Hayat et al. (2021:7), who found that students and lecturers were not satisfied with the slow internet speed, resulting in slow uploading and downloading in the LMS. The situation was worsened by electric power outages, which affected network signals. Consequently, classes had to be postponed to other days, resulting in increased workloads for the lecturers. Infrastructural challenges cause severe frustration for the lecturers because they do not have any control over them (Baroudi & Shaya 2022:13; Zhu & Zhang 2022:2398).

Although the implementation of online teaching was challenging, there were some benefits for the lecturers. The lecturers were able to reach a larger number of students at a time than they would in traditional face-to-face teaching because the physical teaching space was not a challenge. Online teaching enabled the lecturers to keep in touch with the learners outside the physical classroom and provided an alternative measure for the completion of the curriculum (Saha et al. 2022:5). Furthermore, it was perceived as cost-effective for the lecturers because it reduced travelling cost to the university and was convenient as they held classes from their homes.

The lecturers appreciated learning new online skills required for effective teaching. They had to be creative when preparing and offering classes. Furthermore, they acknowledged the need to be technologically savvy to be able to move with the Fourth Industrial Revolution (4IR). The 4IR is the current and developing situation where changes in technologies and trends are transforming the manner in which people live and work. Therefore, digital literacy is a requirement for lecturers to develop competencies to participate in the global digital society (Kayembe & Nel 2019:87).

The lecturers acknowledged the importance of online teaching and suggested measures that could facilitate the smooth delivery of online teaching. They recognised the need for continuous training and support on how to effectively use the system to translate knowledge and skills to students. Training, together with mentoring by experienced and competent individuals, will prepare the lecturers with the technological knowledge and skills required to design online learning activities that will increase students’ engagement, consequently increasing knowledge and skill translation to learners (Baroudi & Shaya 2022:15).

### Limitations of the study

The study was conducted at a public university in Gauteng, South Africa, which has a small number of nurse lecturers. Therefore, the findings may not be applicable to other universities in the same province.

#### The implications for nursing education

This study aimed to gain an in-depth understanding of nurse lecturers’ experiences with online teaching during the COVID-19 pandemic. These experiences are related to teaching nursing online which is traditionally known to be a face-to-face course. The nurse lecturers’ experiences indicated the need for online teaching to be planned. The plan must include sufficient training for the nurse lecturers and an environment that will support the execution of all teaching aspects of the course. Ongoing support is crucial to the success of online teaching in nursing, including identifying creative ways of delivering clinical content online.

## Conclusion

The study explored nurse lecturers’ experiences with online teaching at a public university in Gauteng, South Africa. The findings reflected a lack of lecturers’ training and support in the implementation of online teaching in nursing. The lack of training and support on the LMS resulted in lectures taking more time to plan for lessons, which caused frustration and discomfort. The need to teach clinical skills online compelled lecturers to be innovative in demonstrating clinical procedures. However, online assessments of clinical competencies remained a challenge. In addition, the inconsistent supply of electricity and unstable internet connectivity was detrimental to the successful implementation of online teaching. Despite these challenges, the borderless benefits of online teaching could assist in enhancing student and staff motivation and retention. These platforms may allow learners and lecturers to work in their areas of comfort.
